# Pediatric Bipolar Disorder: Evolution in Clinical and Biological Markers and Future Perspectives

**DOI:** 10.2174/1570159X2106230410111947

**Published:** 2023-05-12

**Authors:** Kirti Saxena, Kiki Chang, Gabriele Sani

**Affiliations:** 1 Menninger Department of Psychiatry and Behavioral Sciences, Baylor College of Medicine, Houston, Tx, USA;; 2 Private Practice, Palo Alto, CA, USA;; 3 Department of Neuroscience, Section of Psychiatry, Fondazione Policlinico Universitario Agostino Gemelli IRCCS, Rome, Italy

Bipolar disorder BD is a devastating, lifelong illness characterized by frequent mood episodes, increased suicide risk, morbidity, and functional impairment [1]. Retrospective interviews of a large sample of adults with BD indicate that approximately 30% experienced very early onset of their symptoms age 13 or younger, and approximately 40% experienced early onset age 13-18 [2]. According to the US National Comorbidity Survey Adolescent Supplement NCS-A, an estimated 2.9% of adolescents had BD at some time in their lives [3]. Over the past two decades, it has become clear that BD is also a significant problem in children and adolescents [4-6]. Indeed, the literature supports the existence of a developmental continuum for BD across the lifespan, with early-onset cases constituting a particularly severe and genetically loaded form of the illness [7, 8].

Although it is now well established that chronic irritability per se does not constitute a sufficient criterion to define pediatric BD PBD [9, 10], the identification of clear-cut onsets and offsets of distinct manic or depressive episodes in youth is often challenging [11]. Additionally, the frequent comorbidity of PBD with attention-deficit/hyperactivity disorder, oppositional-defiant disorder, substance use, and anxiety disorders adds to the complexity of accurate diagnoses and subsequent course of treatment of the illness [12]. Thus, it is important to study clinical and biological pathways in those youth affected by PBD to aid in diagnosis and understanding of etiology. Equally important is learning how the illness manifests and progresses in those at high risk for developing PBD to be able to eventually the progression to such a complex and impairing disorder.

This thematic issue on PBD appraises the readers of new research and systematic reviews on clinical, pharmacological, neural and biological aspects of PBD. It provides further insight into youth at high risk for developing the illness.

Apicella et al. broach the topic of gender differences in the psychopathology of mixed depression in adolescents and highlight differences in clinical symptoms amongst males and females. In clinical settings, these aspects assist clinicians in looking for the different ways depression can manifest.

While recognizing clinical symptoms of mania and depression in youth is indeed important, knowing who is at high risk for developing PBD and intervening early may be even more impactful. Towards this end, Miklowitz et al. explored psychosocial and neuroimaging variables as mediators of treatment effects in youth at high risk for developing PBD.

It is challenging to perform controlled clinical pharmacological trials in youth with PBD. Assessing the effectiveness of pharmacological treatments in clinical settings remains a challenge but is warranted, and the long-term effects of psychotropics on the course of the illness are sorely needed. Although the use of lithium in treating adult bipolar disorder is well recognized, there is limited data on the use of lithium in PBD. Janiri et al. provide a systematic review of the use of lithium in PBD, supporting its use in PBD, albeit with limited data. Notably, targeting the specific symptoms in different mood states can be impactful. For instance, helping a child sleep well can positively impact mood, attention, and energy. Thus, Singh et al. show that cardinal symptoms, such as sleep problems and irritability, in youth with bipolar depression can influence treatment outcomes. Also, as many youths are prescribed antipsychotics for treating PBD, studying how these medications affect metabolic parameters differently in males and females is relevant. Ilzarbe et al. walk readers through a systematic review and meta-analysis of sex differences in serum prolactin levels in youth taking antipsychotics.

Emotional dysregulation in youth has been related to several neurocognitive alterations, including processing biases towards positively or negatively balanced emotions [13-15]. Functional and structural magnetic resonance imaging techniques have shown that youth with PBD endorsed primary alterations in networks involving areas related to mood control, such as the amygdala, the hippocampus, the VLPFC, the DLPFC, and the anterior cingulate [16]. However, recent findings suggest that the neural basis of altered mood regulation extends beyond the affective network and includes several interrelated networks and areas that involve cognition and movement [17, 18]. In a new research article, Saxena et al. discuss the relationship between cerebellar volumes and cognitive domains in youth with PBD and bipolar offspring. Luciano et al., furthermore, review the structural and functional brain characteristics associated with PBD. Finally, using another dimension to understand brain functional abnormalities in PBD, Zou et al. discuss their findings on examining brain temperature and its relation to cerebral blood flow in youth with PBD.

This thematic issue contributes to the ongoing efforts of scientists dedicated to discovering and understanding the clinical and pathophysiological underpinnings of pediatric bipolar disorder and those at high risk for developing the illness. Continued work in this area will help to push the field forward to identify biological markers that can be used to both help correctly diagnose and aid in the treatment of youth with PBD and even towards early identification and prevention of the more fully formed disorder that likely affects millions of youth worldwide.
